# Palmitoylethanolamide Supplementation during Sensitization Prevents Airway Allergic Symptoms in the Mouse

**DOI:** 10.3389/fphar.2017.00857

**Published:** 2017-12-12

**Authors:** Fiorentina Roviezzo, Antonietta Rossi, Elisabetta Caiazzo, Pierangelo Orlando, Maria A. Riemma, Valentina M. Iacono, Andrea Guarino, Armando Ialenti, Carla Cicala, Alessio Peritore, Raffaele Capasso, Vincenzo Di Marzo, Angelo A. Izzo

**Affiliations:** ^1^Department of Pharmacy, School of Medicine, University of Naples Federico II, Naples, Italy; ^2^Institute of Protein Biochemistry, National Research Council, Naples, Italy; ^3^Institute of Applied Sciences and Intelligent Systems, National Research Council, Naples, Italy; ^4^Endocannabinoid Research Group, Naples, Italy; ^5^Institute of Biomolecular Chemistry, National Research Council, Naples, Italy; ^6^Department of Agricultural Sciences, University of Naples Federico II, Portici, Italy

**Keywords:** palmitoylethanolamide, mast cells, allergen sensitization, bronchial hyperreactivity, airway inflammation

## Abstract

One important risk factor for the development of asthma is allergen sensitization. Recent increasing evidence suggests a prominent role of mast cells in asthma pathophysiology. Since Palmitoylethanolamide (PEA), an endogenous lipid mediator chemically related to – and co-released with- the endocannabinoid anandamide, behaves as a local autacoid down-regulator of mast cell activation and inflammation, we explored the possible contribution of PEA in allergic sensitization, by using ovalbumin (OVA) as sensitizing agent in the mouse. PEA levels were dramatically reduced in the bronchi of OVA-treated animals. This effect was coupled to a significant up-regulation of CB_2_ and GPR55 receptors, two of the proposed molecular PEA targets, in bronchi harvested from allergen-sensitized mice. PEA supplementation (10 mg/kg, 15 min before each allergen exposure) prevented OVA-induced bronchial hyperreactivity, but it did not affect IgE plasma increase. On the other hand, PEA abrogated allergen-induced cell recruitment as well as pulmonary inflammation. Evaluation of pulmonary sections evidenced a significant inhibitory action of PEA on pulmonary mast cell recruitment and degranulation, an effect coupled to a reduction of leukotriene C_4_ production. These findings demonstrate that allergen sensitization negatively affects PEA bronchial levels and suggest that its supplementation has the potential to prevent the development of asthma-like features.

## Introduction

Asthma is a common disease which affects around 5% of the global population. For the majority of patients symptoms can be controlled with a combination of inhaled corticosteroids, bronchodilators and LT modifiers (Wilson et al., 2006; Lang, 2015). However, between 5 and 10% of patients have asthma that is refractory to current treatment (Kudo et al., 2013). As such, a greater understanding of the underlying immune mechanisms leading to and perpetuating the asthmatic condition are required in order to develop new effective therapeutic approaches (Umetsu et al., 2002; Kim et al., 2010).

The pathophysiology of allergic asthma is multifaceted and characterized by airway inflammation and inflammatory cell infiltration (e.g., eosinophils), that lead to alteration of airway physiology, manifesting as variable airflow obstruction and airway hyperreactivity. In recent years attention has been increasingly switched to the role of mast cells in the pathophysiology of the asthmatic process (Galli and Tsai, 2012). For a long time mast cells have been considered critical only for the expression of immediate allergic reactions, such as anaphylaxis or the acute wheezing provoked by allergen challenge in subjects with atopic asthma; however, it has now become evident that these cells contribute to the expression of both IgE-dependent late phase reactions and some important features of chronic allergic inflammation (Galli et al., 2005; Bulfone-Paus and Bahri, 2015). In particular, mast cells participate in the initiation of such responses and can function as immunoregulatory cells. Indeed, a number of environmental factors cooperate to determine the type, magnitude or duration of mast cell secretory responses. Within seconds of activation, mast cells release a multitude of preformed biologically active products, followed by marked changes in their cytoplasmatic composition. Via the secretion of these mediators, mast cells participate in the sensitization phase of the acquired immune response sustaining dendrite cell maturation, function and recruitment to local lymph nodes (Oskeritzian, 2015). Mast cells also produce many cytokines that recruit eosinophils, forming an “allergic effector unit” that contributes to allergic disorders (Galdiero et al., 2017). However, mast cells also exert important functions by interacting with airway structures and influencing airway function (Bradding and Arthur, 2016; Virk et al., 2016).

Very little is known about the endogenous molecules and mechanisms capable of modulating mast cell activation. PEA is both a naturally occurring lipid ingredient contained in foods/dietary supplements and an endogenous lipid mediator belonging to the class of fatty acid ethanolamides (Mattace Raso et al., 2014; Iannotti et al., 2016). PEA is biosynthesized “on demand” from membrane phospholipids (Cadas et al., 1996; Cravatt et al., 1996; Ueda et al., 2013) and has been proposed to behave as a local autacoid mediator able to down-regulate mast cell activation and inflammation (Aloe et al., 1993). Evidence indicates that PEA is an important anti-inflammatory, analgesic and neuroprotective mediator acting on several molecular targets in both central and in peripheral organs and systems (Iannotti et al., 2016). Direct or indirect (via effects on endogenous ligand levels or receptor expression) molecular targets for PEA include PPAR-α, TRPV1 channels, cannabinoid (CB_1_ and CB_2_) receptors and GPR55 (De Petrocellis et al., 2004; LoVerme et al., 2005; Ryberg et al., 2007; Ho et al., 2008; Pertwee et al., 2010). PEA metabolism is disturbed during inflammation, pain and neurodegeneration, resulting in an increase or decrease in its levels in tissues most affected by the disease (Alhouayek and Muccioli, 2014; Mattace Raso et al., 2014; Iannotti et al., 2016).

In the light of the well-established role of mast cells in asthma pathogenesis and considering that PEA inhibits mast cell recruitment and degranulation, we have evaluated its role in allergen sensitization and inflammation.

## Materials and Methods

### Materials

Ultramicronized PEA (powder particle size <10 μm, with the following distribution: <6 μm, 99.9%; <2 μm, 59.6%; <1 μm, 14.7%; <0.6 μm, 2%, as described in patent EP2475352 A1, with text from patent WO2011027373A1) was kindly provided by Epitech Group (Saccolongo, Italy). All other reagents and fine chemicals were obtained from Sigma–Aldrich (Milan, Italy).

### Animals

The animal studies are reported in accordance with the ARRIVE guidelines for reporting animal research (Kilkenny et al., 2010). Female BALB/c mice (18 ± 2 g body weight, Charles River, Calco, Italy) were housed in a controlled environment (21 ± 2°C) and provided with standard rodent chow and water. All animals were allowed to acclimate for 4 days prior to experiments and were subjected to 12 h light – 12 h dark schedule. Experiments were conducted during the light phase. The experimental procedures, according to Italian (DL 26/2014) and European (n. 63/2010/UE) regulations on the protection of animals used for experimental and other scientific purposes, were approved by the Italian Ministry (Number 1092/2015).

### Sensitization and Drug Treatment

**FIGURE 1:**
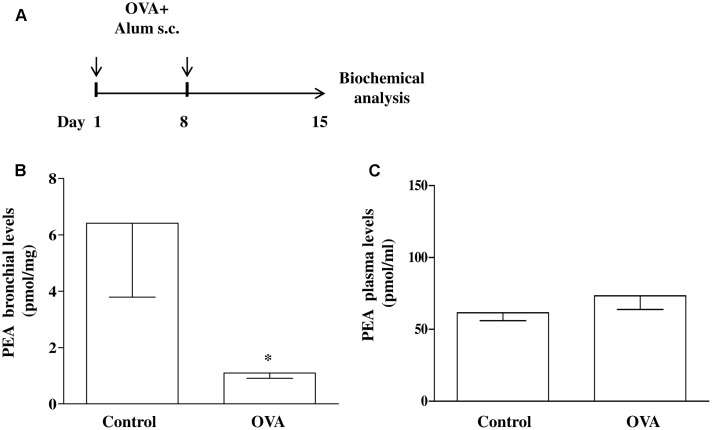
PEA levels in OVA sensitized mice. **(A)** Mice were injected with 0.4 ml s.c. of a suspension containing 100 μg of OVA absorbed to 3.3 mg of aluminium hydroxide gel (OVA) or vehicle (control) on days 1 and 8. Bronchi were harvested and analyzed 15 days after vehicle or OVA administration. PEA levels in the bronchi **(B)** and in plasma **(C)** were measured by liquid chromatography-mass spectrometry and expressed as pmol/mg tissue and pmol/ml, respectively. Data are expressed as mean ± SEM, *n* = 6 animals for each group; ^∗^*P* < 0.05 vs. control; one-tailed Student *T*-test.

Mice were injected subcutaneously (s.c.). with 0.4 mL of a suspension containing 100 μg of OVA absorbed to 3.3 mg of aluminium hydroxide gel on days 1 and 8 (OVA-sensitized mice), while control animals received an equal volume of vehicle (**Figure 1A**) (Roviezzo et al., 2007, 2015; Sullo et al., 2013). Mice were sacrificed at 15 days after OVA administration to take the bronchi and blood to evaluate PEA levels and metabolism.

In another set of experiments PEA (10 mg/kg; OVA+PEA) (Impellizzeri et al., 2013; Capasso et al., 2014) or vehicle (saline, ETOH and Tween-20, 8:1:1, by volume; OVA) were administered intraperitoneally (i.p.) 15 min before each OVA administration. Mice were euthanized 15 (**Figure 5A**) and 22 (**Figure 3A**) days after OVA sensitization and main bronchi and lungs were collected and processed as described below.

### Identification and Quantification of Plasma and Bronchial PEA

Main bronchi and blood were harvested from mice, 15 days after OVA or vehicle administration, and immediately immersed into liquid nitrogen and stored at -80°C until extraction of PEA. PEA was extracted from 100 μL of plasma by liquid–liquid extraction and separated by liquid chromatography (Ultimate 3000 RS, Dionex, CA, United States). Quantitative analyses were performed on a 5500 QTrap^®^ triple quadrupole/linear ion trap (QqQLIT) mass spectrometer equipped with a TurboIon-Spray TM interface (AB Sciex, Concord, ON, Canada).

Tissues were extracted with chloroform/methanol (2:1, by volume) containing 10 pmol of d4-PEA (provided by Cayman Chemicals, United States). The lipid extracts were purified by silica column chromatography and the fractions containing PEA were analyzed by isotope dilution liquid chromatography–atmospheric pressure chemical ionization mass spectrometry (LC-MS). Results were expressed as picomoles per milligram of tissue (Borrelli et al., 2015).

### Quantitative (Real-Time) RT-PCR Analysis

The bronchi from mice treated with vehicle (control group) or OVA were removed (15 days after the administration of OVA or vehicle), collected in RNA later (Invitrogen, Carlsbad, CA, United States) and homogenized by a rotorstator homogenizer in 1.5 mL of Trizol^®^ (Invitrogen). Total RNA was purified, quantified, characterized and retrotranscribed as previously described (Grimaldi et al., 2009). For all samples tested, the RNA integrity number (Bioanalyzer 2100, Agilent) was greater than eight relative to a 0–10 scale. Quantitative real-time PCR was performed by an iCycleriQ5^®^ (Bio-Rad, Milan, Italy) in a 20 μL reaction mixture as described. Assays were performed in quadruplicate (maximum Δ*C*t of replicate samples <0.5), and a standard curve from consecutive fivefold dilutions (100–0.16 ng) of acDNA pool representative of all samples was included for PCR efficiency determination. Optimized primers for SYBR Green analysis and optimum annealing temperatures were designed by the Allele-Id software version 7.0 (Biosoft International, Palo Alto, CA, United States) and were synthesized (HPLC purification grade) by MWG-Biotech (Ebersberg, Germany). For each target, all mRNA sequences at http://www.ncbi.nlm.nih.gov/gene/ were aligned and common primers were designed. Relative expression calculation, correct for PCR efficiency and normalized with respect to reference genes β-actin and hypoxanthine-guanine phosphoribosyltransferase, was performed by the iQ5 software. Results are expressed as fold expression (Romano et al., 2016). Statistical significance was evaluated by the by the REST 2009 software (Pfaffl et al., 2002).

### Bronchial Reactivity

Main bronchi, collected from mice 22 days after sensitization, were rapidly dissected and cleaned from fat and connective tissue. Rings of 1–2 mm length were cut and mounted in 2.5 mL isolated organ baths containing Krebs solution, at 37°C, oxygenated (95% O_2_ and 5% CO_2_), and connected to an isometric force transducer (type 7006, Ugo Basile, Comerio, Italy) associated to a Powerlab800 (AD Instruments). Rings were initially stretched until a resting tension of 0.5 g was reached and allowed to equilibrate for at least 30 min during which tension was adjusted, when necessary, to a 0.5 g and bathing solution was periodically changed. In each experiment bronchial rings were previously challenged with acetylcholine (10^-6^ M) until a reproducible response was obtained. Subsequently, after tissue washing, a cumulative concentration response curve to carbachol (10^-9^ – 3 × 10^-6^ M) was performed. Carbachol–induced contractions were also measured in presence of PEA 10^-5^ M (Romano and Lograno, 2012), administered in the organ bath 30 min before carbachol. Results were expressed as dine *per* mg tissue.

In another set of experiments OVA-sensitized mice were sacrificed at 15 days to take pulmonary tissues and blood for biochemical studies and IgE evaluation, respectively. Plasma IgE levels were measured by means of ELISA using matched antibody pairs (BD Pharmingen, Franklin Lakes, NJ, United States). Each lung was divided into two parts. One part was frozen at -80°C and subsequently homogenized for cytokine and LTC_4_ measurements by ELISA, and the other was fixed in 10% neutralized buffered formalin for histological analysis. Levels of cytokines and LTC_4_ were expressed as pg/mg of tissue.

### Lung Histology

Lung sections were cut (7 μm thick) and stained with H&E for morphological analysis. Mast cell degranulation was evaluated by toluidine staining, following the method described by Iuvone et al. (1999) with some modifications. In brief, it was calculated the percentage of red purple/violet stained cells, i.e., degranulated mast cells, on the total number of mast cells, per mm^2^. Non-degranulated mast cells appeared dark blue stained. Sections were analyzed by blinded operators using a standard light microscope (20× magnification, for H&E staining, and 40 and 100× magnification, for toluidine blue staining) and photographed under low power. Images were taken by a Leica DFC320 video-camera (Leica, Milan, Italy) connected to a Leica DM RB microscope using the Leica Application Suite software V.4.1.0.

### Air Pouch Model

Mice, sensitized as described above, received on days 9 and 12 on the shaved dorsal surface, 2.5 mL s.c. of air to initiate the development of the air-pouches as described previously (Das et al., 1997) (**Figure 4A**). On day 15 (6 days after the first air injection) animals were challenged by injection into the air-pouch with 0.4 mL of sterile saline alone (control group) or containing 10 μg OVA. At different time-points (2 h or 24 h) after OVA or saline injection into the air-pouch, mice were sacrificed by exposition to CO_2_. Air-pouches were washed with 1 mL phosphate-buffered saline (pH = 7.4). Lavage fluids were centrifuged at 300 × *g* for 10 min at 4°C. Supernatants were then collected and stored at -80°C until assayed for IL-4 and IL-13 evaluation by ELISA kits according to manufacturer’s instructions. Levels were expressed as pg/mL. Cell pellets were suspended in phosphate-buffered saline and total cell counts were performed by optical microscopy following Trypan blue staining.

### Statistical Analysis

Data are expressed as mean ± SEM of *n* observations, were *n* represents the number of animals. Statistical analysis has been performed by using one- or two-tailed Student *T*-test, one- or two-way ANOVA for multiple comparisons followed by Bonferroni’s post test (GraphPad Prism 5.0 software; San Diego, CA, United States). Data were considered statistically significant when a value of at least *p* < 0.05 was achieved.

## Results

### PEA Levels Are Reduced in Airways Following OVA Sensitization

Increasing evidence suggest that mast cells critically contribute to sensitization mechanisms, but very little is known about endogenous molecules and mechanisms capable of modulating mast cell activation. We have already established that mast cell activation during allergen sensitization is responsible for significant changes in bronchial reactivity (Roviezzo et al., 2015). Although it has been demonstrated that endogenous PEA levels are reduced an inflammatory environment (Capasso et al., 2001; De Filippis et al., 2010; Bandiera et al., 2014), the level of reduction has not been formally tested in airways. To demonstrate the extent to which allergic sensitization decreases PEA levels, BALB/c mice were sensitized and PEA levels were measured in bronchi harvested from vehicle- (control) and OVA-treated mice (**Figure 1A**). In bronchi harvested from control mice the amount of PEA was about 6 pmol/mg of wet weight tissue. Following OVA sensitization a significant reduction of PEA levels occurred in the bronchi (**Figure 1B**). Conversely PEA plasma levels remained unaltered by OVA sensitization (**Figure 1C**).

### Pulmonary Expression of CB_2_ and GPR55 mRNA Is Up-Regulated Following OVA Sensitization

**FIGURE 2:**
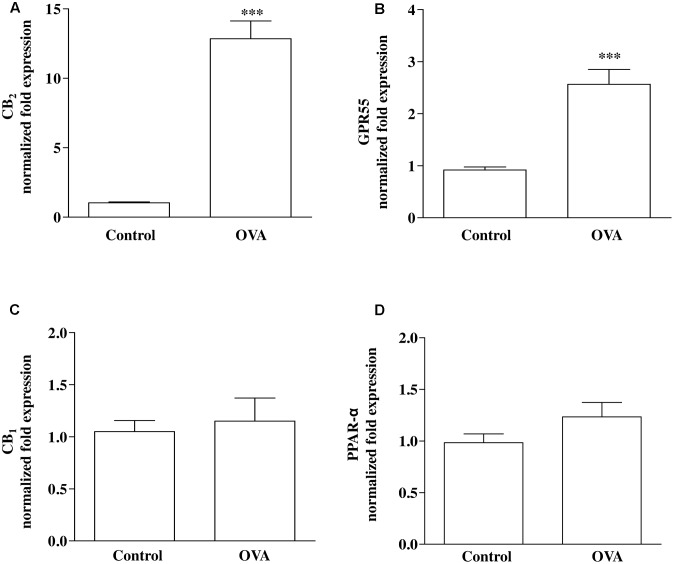
Expression of PEA targets in OVA sensitized mice. Mice were injected with 0.4 ml s.c. of a suspension containing 100 μg of OVA absorbed to 3.3 mg of aluminium hydroxide gel (OVA) or vehicle (control) on days 1 and 8. Bronchi were harvested and analyzed 15 days after vehicle or OVA administration. mRNA expression of **(A)** CB_2_, **(B)** GPR55, **(C)** CB_1_, and **(D)** PPAR-α was evaluated by RT-PCR. Results are calculated as fold expression (mRNA expression). The lower expression values were 32.42 Cq (Control) background N/A, 32.34 Cq (Control) background N/A, 30.60 Cq (Control) background N/A, 27.96 Cq (OVA) background 36.55 Cq for CB_2_, GPR55, CB_1_ and PPAR-α, respectively. Data are expressed as mean ± SEM, *n* = 6 animals for each group; ^∗∗∗^*P* < 0.001 vs. control; two-tailed Student *T*-test.

Direct or indirect (via effects on endogenous ligand levels or receptor expression) molecular targets for PEA include PPAR-α, CB_1_ and CB_2_ receptors, and GPR55 (De Petrocellis et al., 2004; LoVerme et al., 2005; Ryberg et al., 2007; Ho et al., 2008; Pertwee et al., 2010). RT-PCR analysis performed on bronchi harvested from both control and OVA-treated mice showed that CB_2_ (**Figure 2A**) and GPR55 (**Figure 2B**) were up-regulated by OVA sensitization, while CB_1_ (**Figure 2C**) and PPAR-α (**Figure 2D**) were unchanged.

### PEA Prevents OVA-Induced Bronchial Hyperreactivity, But Does Not Affect Bronchial Reactivity *in Vitro*

**FIGURE 3:**
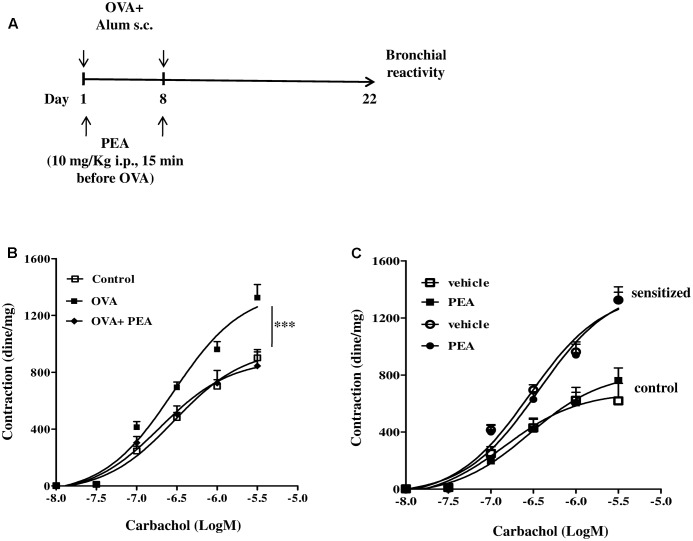
Effect of PEA on OVA-induced bronchial hyperreactivity. **(A)** Scheme of sensitization and drug treatment. Mice were injected with 0.4 ml s.c. of a suspension containing 100 μg of OVA absorbed to 3.3 mg of aluminium hydroxide gel or vehicle (control) on days 1 and 8. After 22 days after OVA sensitization mice were sacrificed. PEA (10 mg/kg) was administered i.p. 15 min before each OVA administration. **(B)** Bronchial reactivity to carbachol was evaluated 22 days after OVA injection. **(C)** Carbachol-induced contractions of bronchi harvested from both control and OVA sensitized-mice in presence or absence of PEA (10^-5^ M). Data are expressed as means ± SEM, *n* = 6 animals for each group. Concentration-response curves **(B,C)** have been generated by a non-linear regression analysis.^∗∗∗^*p* < 0.001; two-ways ANOVA plus Bonferroni.

Palmitoylethanolamide is biosynthesized “on demand” from membrane phospholipids (Cadas et al., 1996; Cravatt et al., 1996; Ueda et al., 2013) and has been proposed to behave as a local autacoid mediator able to down-regulate mast cell activation and inflammation. In order to correlate down-regulation of bronchial PEA to an altered reactivity, we performed functional experiments on bronchi obtained from sensitized mice treated with PEA (10 mg/kg) or with the vehicle (saline, ETOH and Tween-20, 8:1:1,v/v) 15 min before each OVA administration (**Figure 3A**). Bronchi excised from OVA-sensitized mice showed a significant increased reactivity to carbachol, compared to control group and to PEA–treated group (**Figure 3B**).

In another set of experiments, we added PEA in the organ bath and evaluated its effect on carbachol-induced contractions of bronchi harvested from both control and sensitized-mice. In this experimental set up PEA (10 μM) did not exert any effect on bronchial reactivity to carbachol (**Figure 3C**). Thus, PEA effect on bronchial tone is not mediated by stromal cells but its targets might be expressed by infiltrating immune cells.

### PEA Blunts Allergen-Induced Eosinophil Extravasation

**FIGURE 4:**
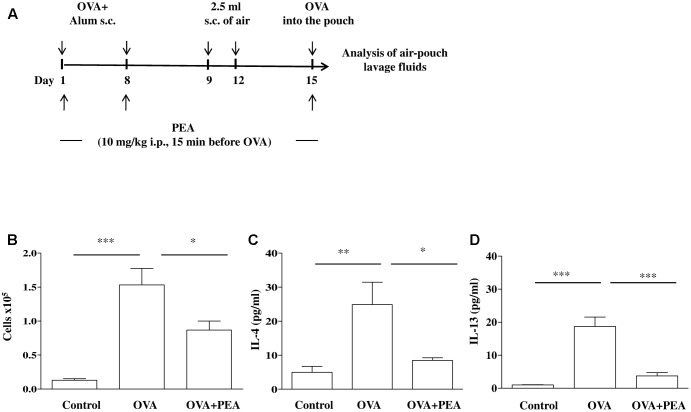
Effect of PEA on OVA-induced inflammation in air pouch. **(A)** Scheme of air pouch model. Animals were injected with 0.4 ml s.c. of a suspension containing 100 μg of OVA absorbed to 3.3 mg of aluminium hydroxide gel (OVA) on days 1 and 8. Then they received at days 9 and 12 on the shaved dorsal surface 2.5 ml s.c. of air. On day 15 mice were challenged by injection into the air-pouch with 0.4 ml of sterile saline alone (control) or containing 10 μg OVA. PEA (10 mg/kg) was administered i.p. 15 min before each OVA administration. **(B)** Cell recruitment, **(C)** IL-4 and **(D)** IL-13 were quantified in lavage fluid of air pouch 24 h (cell recruitment) and 2 h (IL-4 and IL-13) after of OVA challenge, respectively. Data are expressed as means ± SEM, *n* = 5–6 animals for each group; ^∗^*p* < 0.05, ^∗∗^*p* < 0.01, ^∗∗∗^*p* < 0.001; one-way ANOVA plus Bonferroni.

It is a commonly held view that the disordered airway physiology and airway remodeling characteristics of asthma are consequences of airway inflammation and cell infiltration that is typically eosinophilic. In order to assess the role of PEA on eosinophil infiltration we chose the model of allergen-induced eosinophil extravasation into mouse air-pouches (**Figure 4A**) (Das et al., 1997; Rossi et al., 2016). Specifically, air pouch provides a convenient cavity from which cells and mediators can be easily harvested. Injection of OVA into the air pouch of sensitized mice, as described above, provoked an intense allergen-dependent leukocyte infiltration, mainly eosinophilic, as early as 6 h after OVA challenge, with a peak at 24 h (Das et al., 1997). A significant inhibition of cell recruitment triggered by allergen challenge was observed followed pre-treatment with PEA (10 mg/kg; 15 min before OVA administration) (**Figure 4B**). This effect was coupled to PEA ability to prevent OVA-induced increase of both IL-4 and IL-13 cytokine levels (**Figures 4C,D**).

### PEA Blunts OVA-Induced Pulmonary Inflammation

**FIGURE 5:**
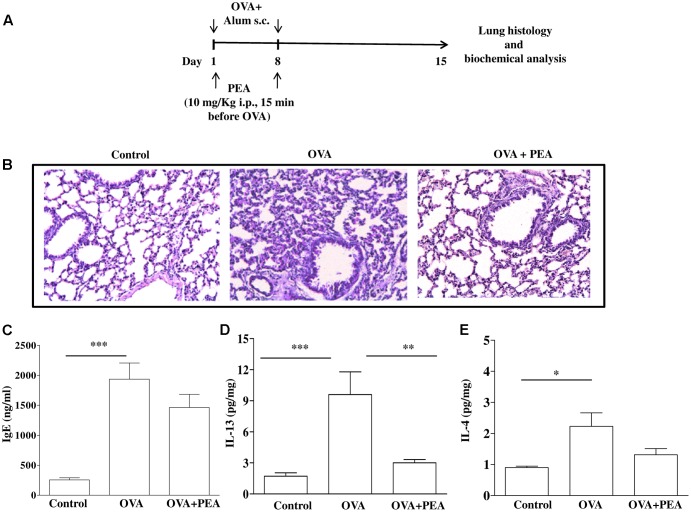
Effect of PEA on pulmonary inflammation induced by OVA sensitization. **(A)** Scheme of sensitization and drug treatment. Mice were injected with 0.4 ml s.c. of a suspension containing 100 μg of OVA absorbed to 3.3 mg of aluminium hydroxide gel or vehicle (control) on days 1 and 8. After 15 days after OVA sensitization mice were sacrificed. PEA (10 mg/kg) was administered i.p. 15 min before each OVA administration. **(B)** H&E staining of lung tissues. **(C)** IgE plasma levels as well as **(D)** IL-13 and **(E)** IL-4 pulmonary levels were quantified by ELISA. Data are expressed as means ± SEM, *n* = 6–10 animals for each group; ^∗^*p* < 0.05, ^∗∗^*p* < 0.01, ^∗∗∗^*p* < 0.001; one-way ANOVA plus Bonferroni.

Since bronchial hyperreactivity is closely related to airway inflammation and PEA displayed a significant inhibition of allergic inflammation, we also evaluated the effect of PEA (10 mg/kg, i.p.; 15 min prior each OVA injection) on the development of inflammation in the lung of allergen sensitized mice. For this reason, mice were sacrificed 15 days following sensitization and pulmonary sections underwent morphological and biochemical analysis (**Figure 5A**). The data obtained demonstrate that PEA prevented pulmonary inflammation; indeed, H&E staining showed reduced peribronchial and alveolar septal inflammatory cell infiltration in the lung of sensitized mice pre-treated with PEA compared to lung sections obtained from OVA-sensitized mice (**Figure 5B**). In order to assess if the beneficial actions of PEA were ascribed to an inhibitory effect on sensitization mechanisms, plasma IgE levels were quantified. The data obtained evidenced that treatment with PEA during sensitization period did not cause any change in plasma IgE levels (**Figure 5C**), but prevented the increase in IL-13 and IL-4 pulmonary levels following sensitization (**Figures 5D,E**).

### PEA Reduces OVA-Induced Mast Cell Activity and LTC_4_ Levels in the Lung

**FIGURE 6:**
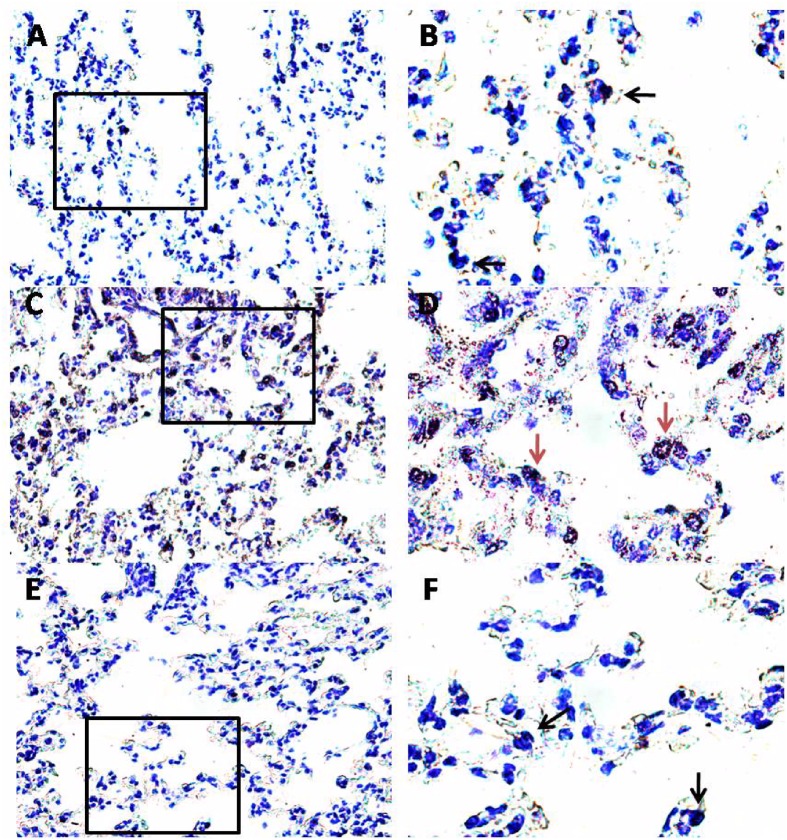
Effect of PEA on pulmonary mast cell recruitment and degranulation induced by OVA sensitization. PEA (10 mg/kg) was administered i.p. 15 min before each OVA administration. Mast cell recruitment and degranulation was evaluated by Toluidine blue staining in pulmonary sections obtained from OVA sensitized mice **(C,D)**, OVA sensitized mice pre-treated with PEA **(E,F)** and control mice **(A,B)**. Quiescent mast cells were dark blue stained (black arrows) while degranulated mast cells were red purple/violet stained (red arrows). **(B,D,F)** Are views at higher magnification (100×) of **(A,C,E)** (40×).

**FIGURE 7:**
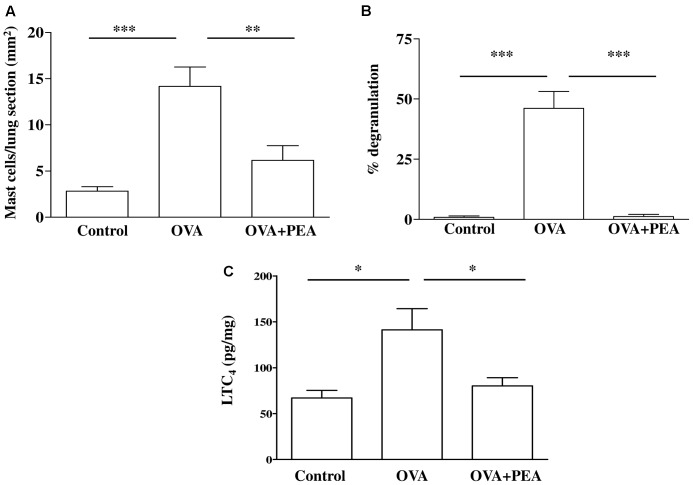
Effect of PEA on pulmonary mast cell activation induced by OVA sensitization. PEA (10 mg/kg) was administered i.p. 15 min before each OVA administration. **(A)** Quantification of mast cell recruitment 15 days after OVA sensitization. **(B)** Percentage of mast cell degranulation evaluated as ratio between degranulated and non-degranulated mast cells. **(C)** LTC_4_ levels were quantified in lung tissue by ELISA. Data are expressed as means ± SEM, *n* = 6; ^∗^*p* < 0.05, ^∗∗^*p* < 0.01, ^∗∗∗^*p* < 0.001; one-way ANOVA plus Bonferroni.

Another key process central in asthma is mast cell recruitment and their localization to different structural components of the airway wall (Galli et al., 2005; Galli and Tsai, 2012). Normal airway epithelium exerts an inhibitory effect on the activity of mast cells, which is subsequently lost in asthma when the epithelium is damaged. As highlighted by toluidine staining (**Figure 6**), OVA sensitization significantly increased mast cell recruitment into the lung, as well as mast cell degranulation (**Figures 7A,B**), effects that well correlate with reduced PEA availability (**Figure 1B**). Conversely, PEA administration (10 mg/kg, i.p.; 15 min prior each OVA injection) prevented both mast cell recruitment and degranulation (**Figures 6**, **7A,B**). LTC_4_ is recognized as the main mediator released by mast cells in allergic asthma; we found that PEA also prevented the increase in LTC_4_ pulmonary levels observed in sensitized mice (**Figure 7C**).

## Discussion

The anti-inflammatory effects of PEA have been for a long time related to its ability to regulate mast cell activation and degranulation, an action that is known as the ALIA mechanism (Aloe et al., 1993). Later on, PEA possible role as a neuromodulator has been proposed by several research groups (Mattace Raso et al., 2014). Compelling evidence indicates that PEA is an important anti-inflammatory, analgesic and neuroprotective mediator acting at several molecular targets in both central and in peripheral organs such as the gut (Capasso et al., 2014; Esposito et al., 2014; Borrelli et al., 2015), the bladder (Pessina et al., 2015), the skin (Vaia et al., 2016), the kidney (Mattace Raso et al., 2013) and the heart (Di Paola et al., 2016). To date, no information has been published on the possible effect of PEA on the respiratory system. Here, we show for the first time that PEA is down-regulated in the airways during allergic sensitization and its exogenous supplementation may prevent many of the asthma-like features.

There is much evidence suggesting that PEA metabolism is disturbed during inflammation (Petrosino and Di Marzo, 2017). Here, for the first time, bronchial PEA levels have been quantified in mice under physiological and atopic conditions (following allergen sensitization). The obtained results show that PEA is present in normal mouse bronchi, its levels (6 nmol/g tissue) being at least 10 times higher than those measured in whole mouse brain (100–550 pmol/g) (Mattace Raso et al., 2014). More importantly and in perfect line with other models of inflammation (Capasso et al., 2001; De Filippis et al., 2010), in the bronchi from sensitized mice the content of PEA was significantly reduced. The down-regulation of PEA levels was specific for the bronchi, since there were no change in plasma PEA levels between control and sensitized mice. Collectively, these data suggest a potentially relevant pro-homeostatic role of this mediator in the airways.

Palmitoylethanolamide acts via a number of targets, including indirect activation of cannabinoid (CB_1_ and CB_2_) receptors and TRPV1 channels, and direct activation of PPAR-α and GRP55 (Petrosino and Di Marzo, 2017), that are all expressed in mouse bronchi. Data on mRNA expression clearly showed that two of such targets, namely CB_2_ and GPR55, were up-regulated in OVA-treated mice. This up-regulation might further suggest an important role of PEA in bronchial homeostasis; indeed, such up-regulation might be seen as an adaptive response able to counterbalance the reduced PEA levels. This is in perfect line with high expression of CB_2_ and GPR55 receptors in immune cells and with their role in driving PEA modulation of immunofunction and inflammation (Zhou et al., 2016).

In parallel to the reduced PEA bronchial levels, OVA-sensitized mice showed increased bronchial reactivity and pulmonary inflammation also in absence of an airway challenge, as previously reported (Roviezzo et al., 2015). In order to understand the significance of the reduced levels of PEA in asthma-like features, we administered exogenously PEA to mice during the sensitization period. For this purpose, mice sensitized and treated with PEA were sacrificed at different time points and bronchial and pulmonary tissues were harvested and used for functional and molecular studies. The evaluation of lung function was performed by measuring bronchial reactivity, which significantly increased following sensitization. Conversely, when sensitized mice were pre-treated with PEA, airway hyperreactivity did not occur. In order to gain further insight into the molecular mechanisms responsible for beneficial actions of PEA, we evaluated its direct effect on bronchial tone, by adding it directly into the organ baths containing bronchial rings. Under these experimental conditions, however, we did not observe any direct effect of PEA on bronchial reactivity, thus suggesting that the effect of PEA on airways is likely due to a specific effect on the infiltrated immune cells. Accordingly, PEA inhibited cell infiltration in a well-known experimental model of allergen-induced eosinophil extravasation into mouse air-pouches. This anti-inflammatory effect was coupled to a reduction of Th2 cytokines, such as IL-4 and IL-13. In addition, PEA showed a beneficial action also on lung inflammation. Indeed, pulmonary cell infiltration of sensitized mice pre-treated with PEA was significantly reduced when compared to untreated sensitized mice. Finally, we ruled out an interference of PEA on sensitization mechanisms, since IgE plasma levels were not modulated by PEA. Thus, it is feasible that PEA modulates the inflammatory immune microenvironment induced by sensitization in the airways, which is consistent with the down-regulation observed in the bronchi, but not in the blood.

Considering the key role of mast cells in asthma pathogenesis and the finding that PEA reduces mast cell recruitment and degranulation *in vitro* (Aloe et al., 1993; Boyce, 2007), we evaluated whether exogenous PEA administration could have any effect on mast cell infiltration/activation. Indeed, pulmonary mast cells not only can act as pro-inflammatory effector cells and drivers of tissue remodeling in established acquired immune responses, but they may also contribute – by acting as immunoregulatory cells - to the initiation and regulation of such responses.

Evaluation of pulmonary sections with Toluidine staining evidenced a significant increase in both mast cell infiltration and degranulation following sensitization. This effect was further confirmed by a significant increased amount of pulmonary LTC_4_, which is recognized as one of the main mediators released by mast cells in allergic asthma (Boyce, 2007). There are some evidences that PEA reduces mast cell activation associated with inflammatory processes. In particular Facci et al. (1995) demonstrated that PEA inhibited, in a concentration-dependent fashion, RBL-2H3 cell degranulation induced by IgE receptor crosslinking and this effect was mediated by CB_2_ agonist activity (Facci et al., 1995). Recently it has been also demonstrated a pivotal role of GR55 for the majority of the inhibiting effects of PEA on human activated mast cells (Cantarella et al., 2011). However, the mechanisms through which PEA is able to regulate mast cell activity has not been completely elucidated. All together these data lead us to hypothesize that upregulation of these receptors in sensitized bronchi was due to an increased mast cell infiltration. Accordingly PEA supplementation prevented mast cell recruitment and degranulation as well as in LTC_4_ pulmonary levels. It is plausible that, in sensitized mice, the decreased levels of PEA represent the loss of an endogenous anti-inflammatory mechanism, in the light of the well-known observation that PEA may behave as local autacoids able to modulate mast cell activation (ALIA mechanism). This hypothesis is supported by other studies demonstrating the therapeutic effect of some anti-inflammatory drugs to be related to a recovery of endogenous PEA levels (Jhaveri et al., 2008).

## Conclusion

Our data demonstrate that an impaired availability of endogenous PEA occurs in the airways in an allergic inflammatory environment. Administration of PEA during the sensitization prevents pulmonary inflammation and the resulting airway hyperreactivity. Since atopy is an important risk factor for asthma we strongly believe that PEA supplementation might have clinical therapeutic effects. In support of our hypothesis: (i) PEA is already available as a nutraceutical for treatment of inflammatory and painful conditions; (ii) PEA has a good safety profile in humans (Petrosino and Di Marzo, 2017).

## Author Contributions

FR, AR, VD, and AAI conceived and coordinated the study and wrote the manuscript. FR, AR, EC, AG, MR, RC, PO, AP, and VI planned and executed the experimental work. CC and AI reviewed and provided scientific input to the manuscript.

## Conflict of Interest Statement

VD is co-inventor of a patent claiming the use of palmitoylethanolamide against pain, and has received research support from Epitech Italia S.r.l., who market palmitoylethanolamide. The other authors declare that the research was conducted in the absence of any commercial or financial relationships that could be construed as a potential conflict of interest.

## Abbreviations

ALIA: autacoid local inflammation antagonism
ANOVA: analysis of variance
CB: cannabinoid
H&E: haematoxylin and eosin
IL: interleukin
LT: leukotriene
OVA: ovalbumin
PEA: palmitoylethanolamide
PPAR: peroxisome proliferator-activated receptor
SEM: standard error of the mean
TRPV1: transient receptor potential vanilloid-type 1.
